# Effects of Thawing and Frying Methods on the Formation of Acrylamide and Polycyclic Aromatic Hydrocarbons in Chicken Meat

**DOI:** 10.3390/foods9050573

**Published:** 2020-05-04

**Authors:** Jong-Sun Lee, Ji-Won Han, Munyhung Jung, Kwang-Won Lee, Myung-Sub Chung

**Affiliations:** 1 Department of Food Science and Technology, Chung-Ang University, 4726 Seodongdae-Ro, Daedeok-Myeon, Anseong-Si 17546, Korea; gpqls3873@naver.com (J.-S.L.); w_w3333@naver.com (J.-W.H.); 2 Department of Food and Biotechnology, Graduate School, Woosuk University, Samnye-eup, Wanju-gun 55338, Korea; munjung@woosuk.ac.kr; 3 Department of Biotechnology, College of Life Sciences and Biotechnology, Korea University, Anam-Dong, Sungbuk-Gu, Seoul 02841, Korea; kwangwon@korea.ac.kr

**Keywords:** chicken, air frying, deep-fat frying, acrylamide, polycyclic aromatic hydrocarbons

## Abstract

Air frying is commonly used as a substitute for deep-fat frying. However, few studies have examined the effect of air frying on the formation of potential carcinogens in foodstuffs. This study aimed to investigate the formation of acrylamide and four types of polycyclic aromatic hydrocarbons (PAHs) in air-fried and deep-fat-fried chicken breasts, thighs, and wings thawed using different methods, i.e., by using a microwave or a refrigerator, or by water immersion. The acrylamide and PAHs were analyzed by high-performance liquid chromatography–tandem mass spectrometry (HPLC-MS/MS) and gas chromatography–mass spectrometry (GC-MS), respectively. Deep-fat-fried chicken meat had higher acrylamide (n.d.–6.19 μg/kg) and total PAH (2.64–3.17 μg/kg) air-fried chicken meat (n.d.–3.49 μg/kg and 1.96–2.71 μg/kg). However, the thawing method did not significantly affect the formation of either acrylamide or PAHs. No significant differences in the acrylamide contents were observed among the chicken meat parts, however, the highest PAH contents were found in chicken wings. Thus, the results demonstrated that air frying could reduce the formation of acrylamide and PAHs in chicken meat in comparison with deep-fat frying.

## 1. Introduction

Although meat consumption has increased steadily in recent years, the consumption of low-fat, low-calorie, and high-protein chicken has grown significantly [1]. The number of chicken franchise stores in Korea has increased from 9000 in 2002 to 24,602 in 2018 [2]. Additionally, the domestic consumption of chicken has continued to grow from 7.5 kg per person per year in 2005 to 14.2 kg per person per year in 2018 [3]. The consumption of chicken in the USA has also increased from 39.2 kg per person per year in 2005 to 42.6 kg per person per year in 2018 [4]. In the European Union, the production of chicken has increased from 8169 billion tons per year in 2005 to 12,260 billion tons per year in 2018 [5]. Furthermore, the most frequently consumed meat cooked at high temperatures is fried chicken [6]. 

Generally, frying involves more rapid heat transfer in comparison with other cooking methods. The lowest temperature employed for frying is 140 °C, although fried food is typically cooked at temperatures between 175 °C and 195 °C [7]. High temperatures promote dehydration of the crust, oil intake, and the chemical reactions of various food constituents such as the denaturation of proteins and the caramelization of carbohydrates [8,9,10]. Moreover, the compounds produced via the Maillard reaction during frying enhance the aroma, color, crust, and texture of the food but reduce its nutritional quality [11,12].

Many epidemiological studies have indicated that a high consumption of processed meats may increase the risk of cancer (e.g., breast, prostate, colorectal, ureter, and pancreatic) in humans because the high cooking temperatures employed in their production can result in high levels of carcinogenic compounds, such as acrylamide and polycyclic aromatic hydrocarbons (PAHs) [13,14,15]. Acrylamide, which is classified as a probable carcinogen to humans (Group 2A) by the International Agency for Research on Cancer (IARC) [16], is also produced during the frying of chicken [14]. Additionally, based on previous studies regarding the carcinogenicity, epidemiology, and mutagenicity of the PAH benzo[a]pyrene (B(a)P), it was categorized by the IARC as a Group 1 carcinogen, indicating that it is carcinogenic to humans [17]. Furthermore, PAHs are known to be endocrine disorder substances, which have long residual terms and exhibit high toxicities as carcinogens [18]. Formerly, the European Commission (EC) regulations required the use of B(a)P as a marker for the content of carcinogenic PAHs in foods [19]. However, as B(a)P was not always found in foods containing PAHs, in 2008, a group of four PAHs (PAH4) and a group of eight PAHs (PAH8) were proposed as better indicators based on data relating to occurrence and toxicity [20]. However, PAH8 measurements provided no additional advantage over PAH4 measurements. Based on comments from the European Food Safety Authority, in 2011, the EC expanded the scope of their regulations to include other food types and to add restrictions on PAH4 levels [21].

As the formation of carcinogenic compounds, such as acrylamide and PAHs, in chicken meat poses a significant risk to human health, studies are required to determine alternative cooking methods that produce healthier products without compromising the texture, flavor, taste attributes, and appearance [22,23,24]. For example, some previous studies have investigated limiting the formation of carcinogenic compounds through the use of precooking methods, such as microwave prethawing, predrying, and low-pressure frying [25,26,27]. An additional means to reduce the formation of carcinogenic compounds is the use of a different cooking method. For example, air frying is commonly used as a substitute for deep-fat frying. This method produces fried food using only a small quantity of fat through oil droplets spread in a hot air stream. Direct contact between the dispersion of oil droplets in hot air and the product inside a closed chamber provides constant heat transfer rates between the air and the food being fried. Thus, this technology permits a reduction of ~90% in the fat content of fried products [24]. Moreover, a 77% reduction in the acrylamide content of air-fried French fries has also been reported (30 μg/kg for air frying and 132 μg/kg for deep-fat frying [28]). Importantly, the air-fried food was crispy on the outside and moist on the inside, and the sensory properties of the final product were similar to those obtained after deep-fat frying. Furthermore, the smell of the food during cooking was less intense in comparison with other frying methods. For this reason, air fryer purchases have risen from 2% in 2014 to 38% in 2018 [29].

However, despite the number of studies conducted to evaluate the effect of hazardous compound formation in fried foods, few studies have examined the effect of air frying on the formation of acrylamide and PAHs in foodstuffs. Thus, we herein investigate the formation of acrylamide and four types of PAHs in chicken breasts, thighs, and wings fried by air frying and deep-fat frying after thawing using a microwave or a refrigerator, or by water immersion. Higher acrylamide and PAH contents were found in deep-fat-fried chicken meat than in air-fried chicken meat, but the thawing method did not significantly affect the formation of either acrylamide or PAHs. Thus, air frying is a promising method for reducing the formation of potentially hazardous compounds in chicken.

## 2. Materials and Methods

### 2.1. Raw Materials

Frozen skinless chicken breasts as well as thighs and wings with skin (Harim Co. Ltd., Iksan-si, South Korea) were purchased from a local food market (Jeonju, South Korea). Soybean oil (CJ CheilJedang, Seoul, South Korea) was purchased from a local food market and used for frying the chicken samples.

### 2.2. Chemicals

Acrylamide (>99%) and ^13^C_3_-acrylamide (>99%) were supplied by Cambridge Isotope Laboratories (Andover, MA, USA). Formic acid (>99%) was purchased from Sigma-Aldrich (St. Louis, MO, USA) and methanol was supplied by J.T. Baker (Phillipsburg, NJ, USA).

Strata-X (200 mg, 6 mL) and Bond Elut AccuCAT (200 mg, 3 mL) solid-phase extraction (SPE) cartridges were purchased from Phenomenex (Torrance, CA, USA) and Agilent Technologies (Santa Clara, CA, USA), respectively. Polyvinylidene fluoride (PVDF) syringe filters were purchased from Futecs Co. (Daejeon, South Korea).

Benzo(a)anthracene (B(a)A), benzo(b)fluoranthene (B(b)F), chrysene (CRY), and B(a)P standards as well as benzo(a)pyrene-d12 (B(a)P-d12) and chrysene-d12 (CRY-d12) internal standards (I.S.) were purchased from Supelco (Bellefonte, PA, USA). Organic solvents, including *n*-hexane, ethanol, and dichloromethane, were purchased from Burdick & Jackson (Muskegon, MI, USA). Potassium hydroxide (>85%) for alkali saponification was purchased from Showa Denko (Tokyo, Japan). Sodium sulfate anhydrous (>99%) for dehydration from Yakuri Pure Chemicals (Kyoto, Japan) and filter paper from Whatman (Maidstone, UK) were used for the dehydration process. Sep-Pak cartridges Agilent Technologies (Santa Clara, CA, USA) were used for SPE. Polytetrafluoroethylene (PTFE) membrane syringe filters from Whatman (Maidstone, UK) and 1 mL syringes from KOREAVACCINE (Ansan-Si, South Korea) were used for the filtration process.

All chemicals used for the determination of acrylamide and the PAHs were of analytical or high-performance liquid chromatography (HPLC) analytical grade.

### 2.3. Thawing Procedures

Chicken thigh, wing, and breast samples of approximately 100 g were employed for the thawing experiments. The frozen chicken samples were subjected to three different home-based thawing practices prior to frying. In accordance with the sanitation standard operating manual [30], the chicken was packed in a low-density polyethylene plastic bag (Cleanwrap, South Korea) for thawing by immersion in water and for refrigeration. Microwave thawing was conducted in a polypropylene microwave-specific container (LocknLock, South Korea). Specifically, the thawing practices used in this study were as follows: (i) Thawing in a microwave (KR-M201BWB; Winia Daewoo, South Korea) at 310 W for 3 min; (ii) thawing in a refrigerator (GC-114HCMP; LG Electronics, South Korea) at 4 °C for 24 h; and (iii) thawing by immersion in distilled water at 20 °C for 1 h (changing the water after 30 min). These thawing practices are suggested by the United States Department of Agriculture (USDA) as safe defrosting methods [31]. Frying was performed immediately after thawing to prevent contamination [30].

### 2.4. Frying Procedures

Deep-fat frying was performed in a domestic electric fryer (model DK-201; Delki, South Korea) with an adjustable temperature up to 190 °C, a 6 L capacity, and a nominal power of 2000 W. The temperature was controlled using a digital thermometer. Hot air frying was conducted using an HD9220 Air Fryer (Royal Philips Electronics N.V., Amsterdam, The Netherlands) with an adjustable temperature up to 200 °C, a 2.2 L capacity, and a nominal power of 1425 W. Approximately 100 g of each sample of the thawed chicken thighs, wings, and breasts were fried at the same time when the temperatures of both fryers reached 180 °C. Deep-fat frying was performed for 10 min using an oil volume of 3.6 L, and the oil was changed thrice for triplicate experiments. Air frying was performed for 35 min, and the samples were flipped once after 20 min without oil spray.

### 2.5. Sample Preparation

All fried chicken parts were cooled to room temperature (approximately 20 °C) after frying and the chicken bone was removed. The deboned skinless chicken breasts and the thighs and wings with skin were homogenized using a Ninja blender (Hai Xin Technology Co. Ltd., Shenzhen, China) for 2 min, and the homogenized samples were stored at 4 °C prior to analysis.

### 2.6. Determination of Acrylamide Content

The analytical method employed for acrylamide determination was based on The Ministry of Food and Drug Safety method with minor modifications [32]. Specifically, each homogenized chicken sample (1 g) was added to water (9 mL) and ^13^C_3_ acrylamide (1 mL, 200 ng/mL) in a 50 mL tube. The tube was shaken at 250 rpm for 20 min, centrifuged (COMBI-514R; Hanil Scientific Inc., Gimpo-si, South Korea) at 3500 rpm for 5 min, and filtered through a 0.45 μm PVDF syringe filter. A Strata-X SPE column was conditioned with methanol (3.5 mL) followed by water (3.5 mL). An aliquot of the sample (1.5 mL) was introduced onto the cartridge followed by water (0.5 mL), and the eluent was discarded. Then, further water (1.5 mL) was passed through the column and the eluent was collected. A Bond Elut AccuCAT SPE column was conditioned using methanol (2.5 mL) followed by water (2.5 mL). Finally, after passing an aliquot (0.5 mL) of the eluate collected from the Strata-X SPE column through the preconditioned Bond Elut AccuCAT SPE column, an aliquot (1 mL) of the eluate from the Strata-X column was introduced onto the Bond Elut AccuCAT SPE column and was collected in 2 mL vials.

High-performance liquid chromatography–tandem mass spectrometry (HPLC-MS/MS) was performed using a Shimadzu 30A HPLC system coupled to a Shimadzu MS8040 MS/MS system (Shimadzu, Kyoto, Japan). The HPLC-MS/MS analytical conditions are outlined in Table 1. The multiple reaction monitoring (MRM) transitions for the quantitation of acrylamide and ^13^C_3_ acrylamide (internal standard) were *m/z* 72 > 55 and *m/z* 75 > 58, respectively.

A calibration curve for acrylamide was prepared in the range of 0.5–200 μg/L. The correlation coefficient was 0.999. Based on the calibration curve parameters, the limit of detection (LOD) and the limit of quantification (LOQ) were calculated as 0.186 μg/kg and 0.562 μg/kg, respectively.

### 2.7. Determination of PAH Content

The PAH content was determined using the method of Lee et al. [33] with modifications. Specifically, each homogenized chicken sample (1 g) was placed in a flat-bottomed 300 mL flask, and then spiked with an internal standard mixture (1 mL, 100 μg/kg of CRY-d12 and B(a)P-d12). The combined *n*-hexane phase was washed with distilled water (3 × 50 mL), dried over Na_2_SO_4_, and concentrated using a rotary evaporator (N-1110, EYELA, Seoul, South Korea) at 37 °C to give a final volume of 2 mL. The samples were then eluted using a Sep-Pak silica cartridge, which was activated with hexane (20 mL). The resulting solution was concentrated to dryness under N_2_ gas at 40 °C, and the residue was redissolved in dichloromethane (1 mL). This solution was passed through a 0.45 μm PTFE membrane filter and transferred into a 2 mL amber screw cap vial. Finally, the PAH contents were analyzed using gas chromatography–mass spectrometry (GC-MS) following a slightly modified standard procedure. GC-MS analyses were performed using an Agilent Technologies 7890A gas chromatograph coupled to an Agilent Technologies 5977B mass spectrometer (Agilent Technologies, Santa Clara, CA, USA). The GC-MS conditions used to analyze the PAH contents are outlined in Table 2. The selected ions employed for the four PAHs and the standards in selected ion monitoring (SIM) mode were as follows: B(a)A (*m/z* **228**, 226, 229), B(b)F (*m/z* **252**, 250, 253), CRY (*m/z* **228**, 226, 229), B(a)P (*m/z* **252**, 250, 253), B(a)P-d12 (*m/z* **264**, 263, 265), and CRY-d12 (*m/z* **240**, 236, 241). The values in bold indicate the quantification ions.

### 2.8. Statistical Analysis

The experimental data were evaluated using the analysis of variance (ANOVA). Using the SPSS software program (IBM Inc., Chicago, IL, USA), significant differences (*p* < 0.05) among the means were determined from triplicate analysis using Duncan’s multiple range test. The differences between the means of the two frying methods were estimated using the *t*-test for independent samples. Values were considered significant at *p* < 0.05.

## 3. Results and Discussion

### 3.1. Acrylamide Contents of Air-Fried and Deep-Fat-Fried Chicken Meat Parts

**Figure 1:**
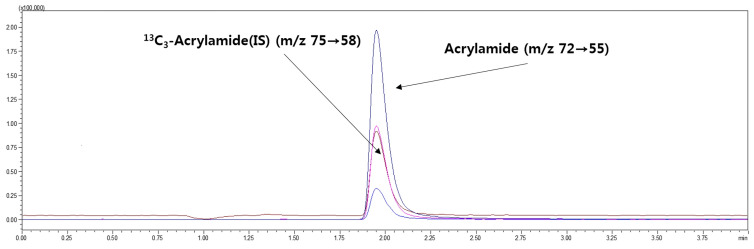
Typical high-performance liquid chromatography–tandem mass spectrometry (HPLC-MS/MS) chromatograms for the acrylamide standard (100 μg/kg) and the internal standard (20 μg/kg) in deep-fat-fried chicken wing samples after thawing by water immersion.

Figure 1 depicts the retention times of the acrylamide standard and internal standard in the HPLC-MS/MS chromatograms of a typical chicken meat sample. The acrylamide contents of the air-fried and deep-fat-fried chicken breast, thigh, and wing samples after thawing in a microwave, in a refrigerator, and by immersion in water are presented in Table 3. The effects of the frying method on acrylamide formation were examined using the same thawing method and the same chicken meat part.

The acrylamide levels of the deep-fat-fried chicken meats ranged from n.d. (not detected) to 6.19 μg/kg, whereas those of the air-fried chicken meats ranged from n.d. to 3.49 μg/kg. Overall, the deep-fat-fried samples contained significantly higher acrylamide levels than the air-fried samples, with the exception of the thigh and wing parts thawed in a refrigerator. For this study, deep-fat frying was performed using soybean oil for 10 min, whereas air frying was conducted without an oil spray for 25 min. Despite the longer frying time, lower acrylamide levels were observed in the air-fried chicken meat samples. It has been proposed that acrylamide is formed from acrolein, which originates from lipid degradation (i.e., oxidized fatty acids or glycerol) [11]. Acrolein is produced when lipids are heated at high temperatures [34]. More specifically, acrolein forms acrylic acid through oxidation and can react to generate an intermediate acrylic radical. Both species can yield acrylamide in the presence of a nitrogen source and under favorable reaction conditions [35]. Similarly, the level of acrylamide formation increased during the heating of potatoes when oil was added [36].

### 3.2. Effect of Thawing Method on Acrylamide Formation

The effect of the thawing method (i.e., microwave, refrigerator, or water immersion) on the formation of acrylamide was examined using the same frying method and the same chicken meat part. No significant differences were observed (*p* > 0.05), except for the deep-fat-fried thigh meat thawed using a microwave. In this context, Erdoǧdu et al. [37] reported that microwave precooking is an efficient way to decrease acrylamide formation in French fries by down-regulating the frying time and temperature. Additionally, Demirok and Kolsarıcı [14] used microwave pretreatment to reduce the levels of acrylamide from 95.15 to 94.13 μg/kg for coated chicken thighs and from 90.47 to 84.38 μg/kg for coated chicken wings.

### 3.3. Acrylamide Formation in Different Chicken Meat Parts

The formation of acrylamide in the chicken breasts, thighs, and wings thawed and fried using the same methods was examined. In general, independent of the frying method employed, the chicken wings contained the highest acrylamide contents, followed by the chicken thighs and the breast meat. No significant differences (*p* > 0.05) were observed among the air-fried chicken meat parts, whereas the deep-fat-fried chicken wings contained significantly higher acrylamide contents than the breast and thigh samples (*p* < 0.05). These results may be due to the different fat contents in the chicken meat parts. The EC established a recommended permitted value of 1000 µg/kg for residual acrylamide in potato crisps [38]; however, in our study, this value was not exceeded for any sample. Specifically, the acrylamide levels of the fried chicken samples produced herein were in the range of n.d.–3.22 μg/kg for the breast samples, n.d.–4.62 μg/kg for the thigh samples, and 2.74–6.19 μg/kg for the wing samples, which are extremely low levels. These results may be due to the use of fresh soybean oil in each frying experiment.

### 3.4. PAH Contents in Air-Fried and Deep-Fat-Fried Chicken Meat Samples

**Figure 2:**
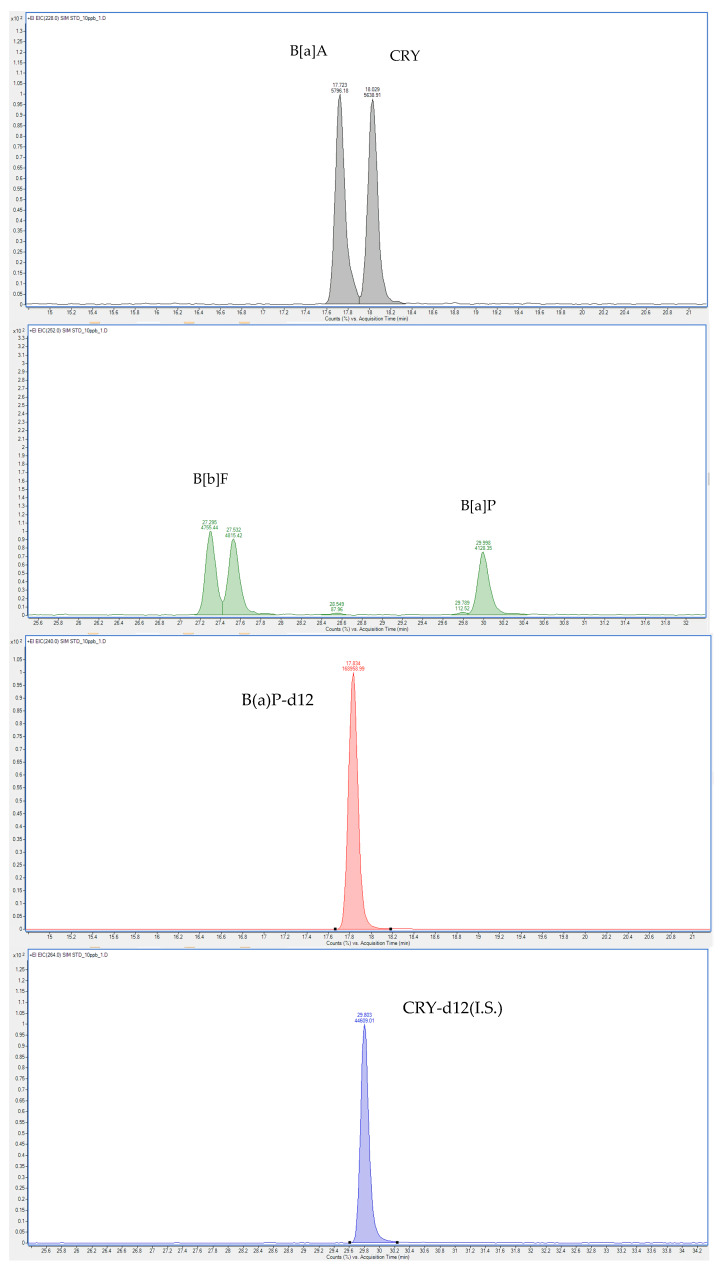
Gas chromatography–mass spectrometry (GC-MS) total ion chromatograms of a mixture of the four polycyclic aromatic hydrocarbons (PAHs) (10 μg/kg) and the B(a)P-d12 and CRY-d12 internal standards (10 μg/kg). Benzo(a)anthracene, B(a)A; benzo(b)fluoranthene, B(b)F; chrysene, CRY; benzo(a)pyrene, B(a)P; benzo(a)pyrene-d12, B(a)P-d12; and chrysene-d12, CRY-d12.

Figure 2 illustrates the GC-MS total ion chromatograms of a standard mixture of the four PAHs (PAH4, 10 μg/kg) and the internal standards (10 μg/kg). The PAH levels of the air-fried and deep-fat-fried chicken breast, thigh, and wing samples after thawing in a microwave, in a refrigerator, or by immersion in water are presented in Table 4. The results obtained for the two frying methods were compared for the same meat parts and thawing methods.

The sums of the four PAH levels for the deep-fat-fried chicken meat samples ranged from 2.60 to 3.17 μg/kg, whereas for the air-fried samples, these values ranged from 1.96 to 2.71 μg/kg. The deep-fat-fried chicken meats exhibited significantly higher (*p* < 0.05) total PAH levels than the air-fried chicken meats. Methyl linoleate is known to produce the highest levels of PAHs, followed by methyl oleate and methyl stearate, and it has been concluded that a greater extent of PAH formation occurs as the degree of unsaturation of the added lipids increases [39]. This behavior was attributed to unsaturated fatty acids being more prone to oxidation during heating [40]. It has previously been discussed that unsaturated fatty acids form cyclic monomers or dimers through polymerization [41]. According to Kostik et al. [42], the unsaturated fatty acid composition of soybean oil consists of oleic acid (28.5%, w/w), linoleic acid (49.5%), and linolenic acid (8%). Additionally, soybean oil should be susceptible to PAH formation because linoleic acid and linolenic acid are likely precursors of such lipid breakdown products [39]. Therefore, our results indicated that the unsaturated fatty acids present in the frying oil promoted PAH production.

### 3.5. Effect of Thawing Method on PAH Formation

The effect of the thawing method (i.e., microwave, refrigerator, or water immersion) on the formation of PAHs was investigated using the same frying method and same chicken meat parts. It was found that the thawing method had no significant effect (*p* > 0.05) on PAH formation in air-fried chicken meats, with the exception of the thigh meat thawed in a refrigerator. In the case of the deep-fat-fried samples, the total PAH levels in the chicken wings thawed in a refrigerator were significantly lower (*p* < 0.05) than those of the chicken wings thawed using either a microwave or water immersion.

Previous research confirmed that thawing in a refrigerator resulted in a lower drip loss of 0.62% among chicken breast samples [43]. Additionally, the presence of water is an important factor in PAH formation because it prevents incomplete combustion by providing oxygen during heating [44,45]. Therefore, it is likely that the lower quantities of PAHs formed in the chicken samples thawed using a refrigerator were due to reduced moisture loss.

### 3.6. PAH Formation in Different Chicken Meat Parts

The formation of PAHs was examined in the chicken breast, thigh, and wing samples that were thawed and fried using the same methods. Specifically, the air-fried chicken wings exhibited the highest PAH contents, followed by the chicken thigh and breast samples. The total PAH levels were in the range of 1.96–2.08 μg/kg for the chicken breasts, 2.08–2.26 μg/kg for the chicken thighs, and 2.31–2.71 μg/kg for the chicken wings. Similar trends were observed for the deep-fat-fried chicken samples. However, no significant differences (*p* > 0.05) were observed among the deep-fat-fried chicken meat samples, with the exception of the chicken breast thawed in the microwave. Lee et al. [33] reported average PAH contents of 0.60–0.76 μg/kg for chicken breasts, 0.94–1.14 μg/kg for chicken thighs, and 0.70–1.17 μg/kg for chicken wings. According to Koh and Yu [46], the lipid contents of chicken wings (14.9%) are higher than those of thighs (2.8%) and breasts (1.2%). Additionally, the most prevalent fatty acid is oleic acid (42.57%), followed by palmitic acid (27.5%) and linoleic acid (15.96%) in chicken meats. Additionally, it has been reported that the fat content of a sample is an important factor in determining the extent of PAH formation in grilled meat [47]. More specifically, PAH formation can occur upon the pyrolysis of organic matter, and the greatest concentrations of PAHs have been shown to arise from the pyrolysis of fat [48], which accounts for the higher levels of PAH formation observed in the chicken wing samples. The regulatory maximum level for B(a)P in smoked meat products is 2 μg/kg, whereas the maximum PAH4 level is 12 μg/kg [21]. In the present study, none of the chicken samples exceeded these maximum values; the B(a)P contents were 0.51–0.79 μg/kg for the chicken breast samples, 0.54–0.78 μg/kg for the chicken thigh samples, and 0.54–0.76 μg/kg for the chicken wing samples. Interestingly, these values were significantly lower than the level of 9.2 μg/kg in duck meat reported by Chen and Lin [49]. However, the B(a)P and PAH4 levels (max 0.79 and 3.17 μg/kg, respectively) were similar to previously reported levels for beef (0.59 and 4.32 μg/kg, respectively) [50]. Furthermore, the maximum B(a)P level fell within the range of n.d.–1.2 μg/kg previously reported for deep-fat-fried chicken breasts [51].

## 4. Conclusions

We herein reported our investigation into the effects of different frying methods (air frying and deep-fat frying) and thawing methods (microwave, refrigerator, and water immersion) of chicken meat parts (breasts, thighs, and wings) on the formation of acrylamide and PAHs. The air-fried samples exhibited lower acrylamide and total PAH contents than the deep-fat-fried samples due to the lower oil content used during the frying process. No significant differences were observed among the thawing methods and the chicken parts in terms of the acrylamide content. However, higher PAH contents were detected in the chicken wing samples in comparison with the chicken thigh and chicken breast samples, likely due to the higher fat content of the chicken wings. Additionally, the amounts of carcinogenic compounds detected in this study were lower than those reported in previous studies because no batter was employed, and the frying oil was relatively fresh. Overall, air frying reduced the formation of acrylamide and PAHs in comparison with deep-fat frying. Therefore, these results may be useful in determining the optimal frying method for chicken in terms of minimizing the formation of potentially hazardous substances.

## Figures and Tables

**Table 1:** Analytical conditions employed for high-performance liquid chromatography–tandem mass spectrometry (HPLC-MS/MS) analysis of acrylamide contents in chicken samples.

Item	Conditions
HPLC instrument	Shimadzu 30A
Column	Kinetex polar C18 (150 mm × 2.1 mm i.d., 2.6 μm particle size, Phenomenex)
Flow rate	0.3 mL/min
Oven temperature	26 °C
Injection volume	20 μL
Mobile phases	0.5% methanol in distilled water and 0.1% acetic acid
MS/MS instrument	Shimadzu MS8040
Ionization mode	Electrospray ionization, 5000 V, positive mode,
Detection mode	MRM mode
Desolvation gas, collision gas	N_2_

MRM: multiple reaction monitoring.

**Table 2:** Gas chromatography–mass spectrometry (GC-MS) conditions for analysis of the four polycyclic aromatic hydrocarbons (PAHs) in chicken samples.

Item	Conditions
GC instrument	Agilent Technologies 7890A
Column	HP-5MS UI (30 m × 250 μm i.d., 0.25 μm film thickness)
Column oven temperature	80 °C (1 min) → 4 °C/min, 220 °C → 20 °C/min, 280 °C (10 min)
Post run	310 °C, 10 min
Flow rate	1.5 mL/min, helium
Injection mode	Splitless mode
Injection volume	1 μL
Injection temperature	320 °C
MS instrument	Agilent Technologies 5977B
Fragmentation mode	Electron impact at 70 eV
Detection mode	SIM mode

SIM: selected ion monitoring.

**Table 3:** Acrylamide levels of air-fried and deep-fat-fried chicken meats thawed using a microwave, a refrigerator, and water immersion.

Frying Method	Thawing Method	Chicken Part	Acrylamide Levels (μg/kg)^1^
Air frying	Microwave	Breasts	n.d.
		Thighs	n.d.
		Wings	3.49 ± 0.54^BXa^
	Refrigerator	Breasts	n.d.
		Thighs	2.23 ± 1.50^AXa^
		Wings	2.84 ± 0.68^AXa^
	Water immersion	Breasts	n.d.
		Thighs	2.10 ± 0.55^BXa^
		Wings	2.74 ± 0.20^BXa^
Deep-fat frying	Microwave	Breasts	n.d.
		Thighs	2.85 ± 0.47^AYb^
		Wings	4.91 ± 0.38^AXa^
	Refrigerator	Breasts	2.52 ± 1.29^AXb^
		Thighs	3.14 ± 0.95^AXYab^
		Wings	4.91 ± 1.87^AXa^
	Water immersion	Breasts	3.22 ± 0.82^AXb^
		Thighs	4.62 ± 0.72^AXb^
		Wings	6.19 ± 1.11^AXa^

^1^: Each value represents the average of three independent repetitions ± standard deviation. ^A, B^ indicate statistically significant differences (*p* < 0.05) between the acrylamide levels of the same thawing methods and the same chicken meat parts where the frying method was varied. ^X, Y^ indicate statistically significant differences (*p* < 0.05) among the acrylamide levels of the same chicken meat parts and the same frying methods where the thawing method was varied. ^a, b^ indicate statistically significant differences (*p* < 0.05) among the acrylamide levels of the chicken meat parts where the same frying methods and same thawing methods were used. n.d. (not detected) indicates that the level was below the limit of detection (LOD).

**Table 4:** Levels^1^ (μg/kg) of the four PAHs in the air-fried and deep-fat-fried chicken breast, thigh, and wing samples thawed in a microwave, in a refrigerator, or by water immersion.

Frying Method	Thawing Method	Chicken Part	B(a)A	B(b)F	CRY	B(a)P	PAH4^2^
Air frying	Microwave	Breasts	0.27 ± 0.03^BXb^	0.28 ± 0.05^AXa^	0.94 ± 0.12^BXb^	0.52 ± 0.03^AXa^	2.00 ± 0.20^BXb^
		Thighs	0.31 ± 0.02^AXb^	0.24 ± 0.05^AXa^	1.06 ± 0.01^BYb^	0.54 ± 0.00^BXa^	2.15 ± 0.07^BXYb^
		Wings	0.43 ± 0.04^AXa^	0.30 ± 0.05^BXa^	1.43 ± 0.10^AXa^	0.55 ± 0.05^AXa^	2.71 ± 0.18^BXa^
	Refrigerator	Breasts	0.26 ± 0.04^AXa^	0.19 ± 0.02^BYb^	0.95 ± 0.05^AXb^	0.56 ± 0.08^AXa^	1.96 ± 0.16^BXb^
		Thighs	0.33 ± 0.03^AXa^	0.20 ± 0.02^BXb^	1.01 ± 0.05^BYb^	0.55 ± 0.02^BXa^	2.08 ± 0.07^BYb^
		Wings	0.32 ± 0.04^AYa^	0.35 ± 0.05^AXa^	1.30 ± 0.08^AXa^	0.54 ± 0.09^AXa^	2.51 ± 0.18^BXa^
	Water immersion	Breasts	0.29 ± 0.02^BXab^	0.25 ± 0.04^AXYa^	1.04 ± 0.08^BXa^	0.51 ± 0.00^BXa^	2.08 ± 0.10^BXa^
		Thighs	0.24 ± 0.04^BYb^	0.22 ± 0.03^AXa^	1.22 ± 0.09^AXa^	0.59 ± 0.05^BXa^	2.26 ± 0.09^BXa^
		Wings	0.32 ± 0.02^BYa^	0.30 ± 0.06^AXa^	1.10 ± 0.11^BYa^	0.59 ± 0.09^AXa^	2.31 ± 0.24^BXa^
Deep-fat frying	Microwave	Breasts	0.42 ± 0.05^AXa^	0.33 ± 0.07^AXYab^	1.30 ± 0.09^AXb^	0.59 ± 0.07^AYa^	2.64 ± 0.14^AXb^
		Thighs	0.39 ± 0.07^AXa^	0.31 ± 0.06^AXb^	1.44 ± 0.14^AXab^	0.76 ± 0.04^AXYa^	2.90 ± 0.2A^Xab^
		Wings	0.46 ± 0.06^AXa^	0.42 ± 0.04^AXa^	1.52 ± 0.06^AXa^	0.76 ± 0.13^AXa^	3.17 ± 0.15^AXa^
	Refrigerator	Breasts	0.35 ± 0.06^AXa^	0.40 ± 0.07^AXa^	1.21 ± 0.24^AXa^	0.64 ± 0.02^AYa^	2.60 ± 0.21^AXa^
		Thighs	0.40 ± 0.03^AXa^	0.35 ± 0.03^AXa^	1.40 ± 0.09^AXa^	0.64 ± 0.02^AYa^	2.79 ± 0.09^AXa^
		Wings	0.40 ± 0.03^AXa^	0.45 ± 0.06^AXa^	1.40 ± 0.09^AXa^	0.59 ± 0.01^AXb^	2.84 ± 0.09^AYa^
	Water immersion	Breasts	0.43 ± 0.05^AXa^	0.27 ± 0.02^AYb^	1.33 ± 0.05^AXa^	0.79 ± 0.03^AXa^	2.81 ± 0.13^AXa^
		Thighs	0.40 ± 0.03^AXa^	0.27 ± 0.04^AXb^	1.33 ± 0.01^AXa^	0.78 ± 0.10^AXa^	2.78 ± 0.08^AXa^
		Wings	0.46 ± 0.03^AXa^	0.36 ± 0.03^AXa^	1.38 ± 0.07^AXa^	0.73 ± 0.09^AXa^	2.93 ± 0.14^AXYa^

^1^: Each value represents the average of three independent repetitions ± standard deviation. ^2^: PAH4 is the sum of the benzo(a)anthracene (B(a)A), benzo(b)fluoranthene (B(b)F), chrysene (CRY), and benzo(a)pyrene (B(a)P) contents. ^A, B^ indicate statistically significant differences (*p* < 0.05) between the PAH levels of the same thawing methods and the same chicken meat parts where the frying method was varied. ^X, Y^ indicate statistically significant differences (*p* < 0.05) among the PAH levels of the same chicken meat parts and the same frying methods where the thawing method was varied. ^a, b^ indicate statistically significant differences (*p* < 0.05) among the PAH levels of the chicken meat parts where the same frying methods and same thawing methods were used.
